# Maternal Blood Lead Levels and the Risk of Pregnancy-Induced Hypertension: The EDEN Cohort Study

**DOI:** 10.1289/ehp.0800488

**Published:** 2009-06-26

**Authors:** Chadi Yazbeck, Olivier Thiebaugeorges, Thierry Moreau, Valérie Goua, Ginette Debotte, Josiane Sahuquillo, Anne Forhan, Bernard Foliguet, Guillaume Magnin, Rémy Slama, Marie-Aline Charles, Guy Huel

**Affiliations:** 1 INSERM, U780, IFR69, Villejuif, France; 2 University Paris–Sud, Orsay, France; 3 Assistance Publique–hôpitaux de Paris, Department of Obstetrics and Gynaecology, University Hospital of Bichat Claude Bernard, Paris, France; 4 Department of Obstetrics and Gynaecology, Regional Maternity, University Hospital of Nancy, Nancy, France; 5 Department of Obstetrics and Gynaecology, University Hospital of Poitiers, Poitiers, France; 6 Avenir Team, INSERM-University J. Fourier Joint Research Center, U823, Institut Albert Bonniot, La Tronche, Grenoble, France

**Keywords:** cadmium, environmental health, epidemiology, gestation, hypertension, lead, manganese

## Abstract

**Background:**

Prior studies revealed associations of environmental lead exposure with risks of hypertension and elevated blood pressure.

**Objective:**

We examined the effect of blood lead levels on blood pressure and the incidence of pregnancy-induced hypertension (PIH) in the second and third trimesters of pregnancy.

**Methods:**

One thousand seventeen pregnant women were enrolled in two French municipalities between 2003 and 2005 for the EDEN (Etude des Déterminants pré et post natals du développement et de la santé de l′ Enfant) cohort study. Blood lead concentrations were measured by atomic absorption spectrometry in mothers between 24 and 28 weeks of gestation.

**Results:**

PIH was diagnosed in 106 subjects (10.9%). Age, parity, weight gain, alcohol, smoking habits, and calcium supplementation were comparable between hypertensive and nonhypertensive women. Lead levels were significantly higher in PIH cases (mean ± SD, 2.2 ± 1.4 μg/dL) than in normotensive patients (1.9 ± 1.2 μg/dL; *p* = 0.02). Adjustment for potential confounder effects slightly attenuated but did not eliminate the significant association between blood lead levels and the risk of PIH (adjusted odds ratio of PIH = 3.3; 95% confidence interval, 1.1–9.7). We also observed geographic differences in lead exposure and in the incidence of PIH and found significant correlations between blood lead levels and unadjusted as well as adjusted systolic and diastolic blood pressures after 24 weeks of gestation.

**Conclusions:**

These findings confirm the relationship between blood lead levels at mid-pregnancy and blood pressure and suggest that environmental lead exposure may play an etiologic role in PIH.

Lead is one of the most extensively studied reproductive toxicants. Several epidemiologic studies have demonstrated a positive association between blood lead levels and blood pressure among nonpregnant adults (Nawrot et al. 2002; Schwartz 1995). The evidence is sufficient to infer a causal relationship of lead exposure with hypertension (Navas-Acien et al. 2007). However, the role of lead in pregnancy-induced hypertension (PIH) remains unclear.

PIH is characterized by an increase in systolic blood pressure (SBP ≥ 140 mmHg) and/or diastolic blood pressure (DBP ≥ 90 mmHg) after 20 weeks of gestation. This disorder can be complicated by proteinuria, a condition corresponding to preeclampsia. PIH is encountered in 10% of pregnancies and is an important cause of morbidity for both mother and fetus (National High Blood Pressure Education Program 2000).

Environmental factors may have a role in this disease occurrence. Although some studies failed to find a relationship between lead concentrations in cord blood and preeclampsia (Angell and Lavery 1982), several authors demonstrated higher blood levels of lead, cadmium, and manganese in hypertensive or preeclampsia patients compared with normotensive women (Dawson et al. 2000; Kosanovic et al. 2002; Rothenberg et al. 1999; Vigeh et al. 2004). Other elements such as zinc and selenium were reported to be reduced in hypertensive pregnant women (Dawson et al. 1999; Rayman et al. 2003).

Blood lead levels increase during pregnancy, from 24 weeks of gestation until delivery, because of increased gastrointestinal absorption and because of an increase in bone turnover in this period (Hertz-Picciotto et al. 2000; O’Flaherty et al. 1995). Several mechanisms may contribute to the pathogenesis of lead-induced hypertension: increases in endothelin and thromboxane production, inhibition of vascular smooth muscle ATPases, oxidation of endogenous nitric oxide by reactive oxygen species, and a decrease in glomerular filtration rate of the kidneys with increase in the renin–angiotensin II–aldosterone activity (Gonick and Behari 2002; Vaziri and Khan 2007; Vaziri and Sica 2004). Interactions between lead and other elements are possible because oxidative stress produced by lead, cadmium, or manganese may be counterbalanced by the antioxidative properties of manganese or selenium (Anastasakis et al. 2008; Campagna et al. 2000; Huel et al. 2000; Vaziri and Sica 2004; Verity 1999).

In the present study, we examined the relationship between PIH and circulating blood lead, cadmium, manganese, and selenium concentrations in a nonselected population of pregnant women.

## Materials and Methods

The study population included the first 1,017 pregnant women enrolled in the EDEN (Etude des Déterminants pré et post natals du développement et de la santé de l**′** Enfant) mother–child cohort study counting 2,002 participants (Drouillet et al. 2008). The number of subjects needed for the study was based on a 10% prevalence of PIH and an estimated relative risk of PIH occurrence of 2. No other factors influenced the inclusion process.

All women 18–45 years of age who presented before 24 weeks of gestation for pre-natal care at two maternity wards in Poitiers (western France) and Nancy (eastern France) were enrolled if they were able to read and write French and were not planning to move out of the region. We also excluded women who had multiple gestations or a history of diabetes. Among women who fulfilled these criteria, 55% agreed to participate. The study was approved by the Ethic Committee of Bicêtre Hospital (November 2002). All partici pants provided informed consent consistent with policies of the INSERM institutional review board. We collected maternal blood samples between 24 and 28 weeks of gestation, just after the recruitment was validated by the study midwife. We determined blood lead, cadmium, and manganese concentrations by electrothermal atomic-absorption spectrometry (model 4100 ZL; Perkin-Elmer, Courtaboeuf, France) with Zeeman background correction as previously described (Fréry et al. 1993; Huel et al. 1986; Mergler et al. 1996). We measured selenium by a standard fluorometric method as described by Lee et al. (1995). We calculated values as means of two analyses of each sample, expressed in micrograms per deciliter. Internal and external quality-control procedures yielded consistently satisfactory results. The limit of detection for the blood lead measurements was 0.5 μg/dL. We assigned a value of 0.5 divided by 2 for values less than the limit of detection. Of the measured values of blood lead, 2.7% were less than the limit of detection.

Gestational age was based on last menstrual period and/or ultrasound-based estimated date of conception. We divided pregnancy into three periods: P1, before 24 weeks; P2, between 24 and 36 weeks; and P3, after 36 weeks of gestation. This choice was mainly founded on scientific data regarding the incidence of PIH in the second half of pregnancy and its frequency with increasing gestational age (Groom et al. 2007).

We measured maternal blood pressure during routine monthly visits, with the subject in supine position, using a standard mercury sphygmomanometer.

One set of measures was taken by the study midwife between 24 and 28 weeks using a different (semiautomated) device, with two measurements averaged to determine the values for that visit.

Measurements taken within each period of gestation (P1, P2, and P3) were averaged to determine the values for SBP and DBP assigned to each period.

Women were classified as having PIH based on SBP ≥ 140 mmHg and/or DBP ≥ 90 mmHg measured during at least two visits after the 22nd week of gestation, such that the elevated measures could have occurred during or across any of the three periods, at any point after 22 weeks. Consequently, no diagnosis of PIH was made before 24 weeks.

We gathered medical and reproductive histories, clinical follow-up, and delivery data from obstetric records. Additional risk factors for PIH were obtained using the study questionnaires presented by trained interviewers to subjects during a structured interview. These included basic socioeconomic information, educational level, and cigarette and alcohol use before and during pregnancy. Dietary information on consumption of coffee and tea and intake of calcium, vitamins, or iron supplements was also recorded.

We evaluated socioeconomic status by the household monthly income and categorized it into three levels (high, > €3,000; medium, €1,500 – €3,000; and low, < €1,500). We also divided education status into two levels (high, ≥ 12 years and low, < 12 years). For the analysis of main effects, all variables (except for hematocrit) were categorized.

Statistical data analysis was performed using SAS software, version 9.1.3 (SAS Institute Inc., Cary, NC, USA). We evaluated variables for normality and for outliers. Because of skewed distributions, lead, cadmium, manganese, and selenium blood levels were transformed into their decimal logarithms and subsequent geometric means were calculated.

Continuous variables were summarized by calculating the mean ± SD. Comparisons of means or proportions were performed by chi-square or Student *t*-tests as appropriate. Cochran-Armitage was used for trend analysis.

We obtained adjusted odds ratios (ORs) by means of multivariable logistic regression analysis with PIH as the dependent variable. We based selection criteria for variables on the literature regarding risk factors of PIH. Besides all elements measured, adjustment variables included maternal age, parity, hematocrit, body mass index (BMI), pregnancy weight gain, gestational diabetes, educational level, socioeconomic status, geographic residence (maternity ward), and smoking status and alcohol consumption before and during pregnancy. Although we used stepwise procedure to ascertain percentage of variance attributable to selected variables, relevant risk factors were forced into the final model. We tested interaction terms between blood lead levels (as a continuous variable) and other maternal variables with a significance level reduced to 0.005 (according to the Bonferroni method). Otherwise, *p*-value < 0.05 was considered to indicate statistical significance.

Pearson partial correlations were calculated between blood lead levels and SBP and DBP during the three periods of pregnancy.

## Results

Among the 1,017 women in the study population, we excluded 31 records (3.0%) because of insufficient sample volumes or analytical problems in metal measurements, and 15 records (1.5%) because of chronic hypertension under treatment before pregnancy. This left a study group of 971 pregnant women. Mothers’ mean age was 29.3 ± 4.9 years. PIH occurred in 106 (10.9%) women and was complicated by proteinuria (preeclampsia) in 20 (2.1%) cases.

In Table 1 we summarize the characteristics of the cohort in relation to PIH occurrence. Compared with the normotensive group, women who developed PIH presented a higher BMI before pregnancy (21.9% vs. 6.3%, *p* < 0.001) and a higher hematocrit level (35.2% vs. 34.6%, *p* = 0.02). They presented more frequently with gestational diabetes (17.0% vs. 5.9%, *p* < 0.001) and premature delivery (13.2% vs. 5.0%, *p* < 0.001). The incidence of PIH was significantly higher in Nancy than in Poitiers (63.2 vs. 36.8% of PIH patients, *p* < 0.001, respectively). PIH was also negatively associated with birth weight. There were very few missing covariate data (< 3.8% in the PIH group and < 5.2% among women without PIH for most of the variables studied).

Mean blood lead concentration in the PIH group (2.2 ± 1. 4 μg/dL; range, 0.2–8.5 μg/dL) was significantly higher than that of normotensive women (1.9 ± 1.2 μg/dL; range, 0.2–6.9 μg/dL; *p* = 0.02). Mean manganese concentration was slightly higher in PIH women but did not reach significance. Cadmium and selenium blood concentrations were comparable between groups (Table 2).

The frequency of PIH was lowest among women whose blood lead concentrations were in the lowest quartile (7.7%) and was significantly greater in the second (10.7%), third (11.1%), and fourth exposure quartiles (13.1%; *p* = 0.03 for trend). The unadjusted OR of PIH associated with an increase of 1 μg/dL in maternal blood lead levels was 3.5 [95% confidence interval (CI), 1.4–8.9].

Adjustment for a range of characteristics and elements (cadmium, manganese, and selenium blood concentrations; hematocrit level; maternal age; BMI; parity; gestational diabetes; education and socioeconomic level; and smoking status and geographic residence) slightly attenuated but did not eliminate the significant association between blood lead levels and the risk of PIH (Table 3). Adjusted OR of PIH for an increase of 1 μg/dL in maternal blood lead levels was estimated at 3.3 (95% CI, 1.1–9.7). We observed no significant interactions among blood lead levels, any of the other elements, and the maternal characteristics in predicting the risk of PIH.

Furthermore, excluding women with preeclampsia (*n* = 20) attenuated the association between blood lead levels and PIH (adjusted OR = 3.1; 95% CI, 1.0–10.0).

When stratified by parity (Table 4), blood lead levels were higher in multiparous than in nulliparous women (2.0 ± 1.2 μg/dL vs. 1.8 ± 1.3 μg/dL, respectively; *p* = 0.003). Adjusted OR for PIH was increased to 4.6 (95% CI, 1.0–21.6) in multiparous compared with 2.9 (95% CI, 0.6–15.7) in nulliparous women. There was no interaction between parity and blood lead (*p* = 0.46).

**Figure 1:**
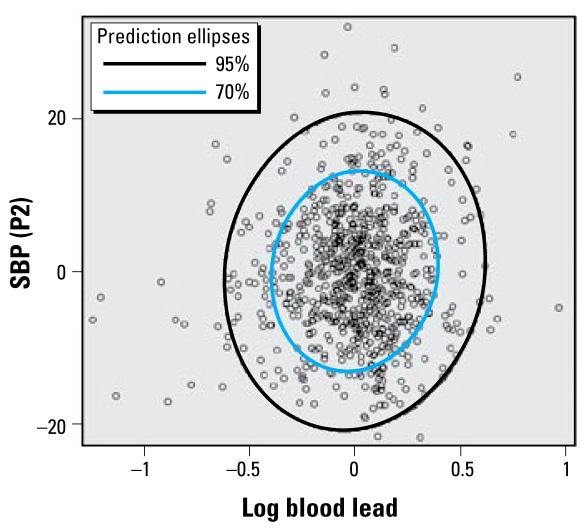
Scatterplot of the residuals for maternal blood lead and SBP between 24 and 36 weeks (P2) after controlling for the effect of variables listed in the logistic model in Table 3: correlation between residuals of the linear regression of the two variables on the partialed variables. In the 95% and 70% prediction ellipses, the major axis length is significantly larger than the minor axis length, indicating a partial correlation between maternal blood lead and SBP at P2.

Log-transformed blood lead at mid-pregnancy was significantly correlated with both SBP (*r* = 0.08; *p* = 0.03) and DBP (*r* = 0.07; *p* = 0.03) after 24 weeks of gestation (P2), and this correlation remained significant after 36 weeks (P3). Figure 1 illustrates correlation between residuals of the linear regression of maternal blood lead and SBP at P2 on the partialed variables. Lead concentrations accounted for approximately 5% of the total unexplained variance obtained by linear models. Each decimal-log increase in blood lead was associated with an increase of 3.5 mmHg in SBP and of 2.5 mmHg in DBP during the second half of pregnancy.

## Discussion

We found that the adjusted risk of PIH was associated with maternal blood lead levels in mid-pregnancy. This risk was doubled in the highest quartile compared with the lowest quartile of lead distribution. There was no strong evidence of an interaction between lead and cadmium, manganese, selenium, or other factors associated with the risk of PIH. The risk of PIH increased with increasing absolute values of mid-pregnancy blood lead in a “dose–response” pattern. A positive correlation was particularly found with SBP at P2 after 24 weeks and persisted after 36 weeks of gestation with SBP at P3. All these findings suggest that blood lead level may be one of the causal factors of PIH.

Evidence has been given that lead increases the circulating levels of endothelin, a vasoactive substance secreted by endothelial cells (Gonick and Behari 2002). Lead is also reported to reduce levels of vasodilator substances such as plasma nitric oxide and endothelial-derived relaxation factor; this reduction is due to a lead-mediated increase in reactive oxygen species (Carmignani et al. 2000; Gonick and Behari 2002; Gonick et al. 1997). Inhibition of membrane ATPases by this metal also leads to increased intracellular calcium ions and vasoconstriction (Moreau et al. 1988). Furthermore, a demonstrable inhibitory effect of lead on blood enzymes such as delta-amino-levulinic acid dehydratase activity was found at a low threshold of lead exposure, ranging from 3.2 to 4.8 μg/dL (Campagna et al. 1999; Telisman et al. 2004). From the above arguments, it may be hypothesized that environmental exposure to lead increases the risk of PIH by inducing vasoconstriction and placental ischemia or by a direct toxicity on the endothelial cell and the renal function.

Our findings are consistent with those from previous studies showing a relationship between blood lead levels and PIH. However, most of these reports analyzed lead concentrations late in pregnancy, from maternal pre-natal red blood cells (Dawson et al. 2000), umbilical cord (Rabinowitz et al. 1987), amniotic fluid (Dawson et al. 1999), or maternal blood within 24 hr after delivery (Vigeh et al. 2004, 2006). Our study did not involve serial measurements of blood lead levels throughout pregnancy. However, the optimal period for such measurement would be in the second trimester (24 weeks of gestation), at the beginning of a potential pathologic change in blood pressure. Moreover, models on lead variation during pregnancy described a constant increase in blood lead levels from 24 weeks of gestation until delivery (O’Flaherty et al. 1995). Indeed, longitudinal analyses showed that maternal blood lead concentrations upon entering prenatal care (average of 13.5 weeks) were not associated with PIH, whereas higher lead levels during the second trimester were related to PIH (Sowers et al. 2002).

The severity of the outcome (i.e., preeclampsia) may be related to the magnitude of lead exposure because we discovered a weaker association between blood lead and PIH when excluding women with preeclampsia. This finding further supports the likelihood of a dose–response relation between blood lead and hypertension.

Our analysis of correlations of SBP at P2/P3 and DBP at P2/P3 with blood lead concentrations was consistent with that of previous studies on essential hypertension (Batuman et al. 1983). Recent reviews suggest a weak but dependable association between lead levels and blood pressure. The increase in SBP is estimated between 0.8 and 2.0 mmHg (Hertz-Picciotto and Croft 1993; U.S. Environmental Protection Agency 2006). We excluded chronic hypertension from our study because of risks of bias associated to prior treatment with antihypertensive drugs. This exclusion might have attenuated the correlation between blood lead and blood pressure.

Manganese blood level was not significantly associated with PIH in this study, although there was some evidence of a possible weak association. The underlying mechanisms of manganese-induced hypertension are probably independent from gestational age. The decrease in blood manganese with intrauterine growth restriction might obscure any other pathologic effect (Vigeh et al. 2008). Cadmium also was not significantly associated with PIH in the present model. This finding may be related to relatively low levels of cadmium observed in this cohort. These reduced levels were probably due to the low prevalence of smoking, which is the main environmental source of cadmium (Bonithon-Kopp et al. 1986). Interaction between selenium and lead concentrations were not significant, and the putative protective effect of selenium through antioxidative properties was not confirmed in this study.

Hematocrit levels are usually elevated in pregnancy hypertensive disorders (Goldstein et al. 2000). This may be due to a lower water balance index correlated to stroke volume or to a lower venous capacitance in hypertensive patients (Aardenburg et al. 2005). Increases in hematocrit automatically increase blood lead, which is concentrated in red blood cells. Although we found no statistical interaction between blood lead and hematocrit, potential confounding can not be ruled out.

BMI was strongly associated with PIH. This finding is consistent with that of previous reports (Hrazdilova et al. 2001; Vigeh et al. 2006). A dose–response relationship has also been observed in this study, so BMI before pregnancy should be considered in all models including PIH. The effect of gestational diabetes on PIH was limited in the multivariate regression analysis probably because of its association with increased BMI.

Age and parity were not significant covariates in this study. Although a trend of increasing blood pressure with age is usually reported for DBP, the restricted age range (18–45 years) and the limited number of women > 40 years of age in the present study may have reduced our ability to highlight an age effect on PIH.

As to parity, high levels of blood lead observed in multiparous women of this study may have contributed to an increase in the frequency of PIH in this group, thus reducing the difference usually observed in the incidence of PIH and preeclampsia depending on parity. Moreover, data obtained from systematic ambulatory monitoring in a large sample of normotensive pregnant women indicate the lack of differences in blood pressure according to parity (Ayala and Hermida 2001).

Finally, PIH frequency was higher in Nancy than in Poitiers. A separate analysis (data not shown) revealed higher blood lead levels in Nancy (2.0 ± 1.3 μg/dL) than in Poitiers (1.8 ± 1.1 μg/dL; *p* < 0.001). Thus, a major difference in the incidence of PIH between these two regions might have been their history of environmental exposure to chemical pollutants. Blood lead level primarily reflects recent exposure, although a part of lead in blood may originate from lead stored in bone, particularly during pregnancy (Gulson et al. 1997). Because bone lead has a half-life of years to decades, the higher blood lead levels observed in eastern versus western region reflect differences in current exposure, but may also reflect some differences in past exposure, or both. Hence, the relationship between blood lead and blood pressure may be mediated by the contribution of bone lead to blood lead, placing a larger amount of bioavailable lead into tissues and organs that affect blood pressure (Rothenberg et al. 1999).

The main sources of measurement errors of blood pressure are the inaccuracy of meas urement methods and the intraindividual variability of blood pressure. We attempted to minimize these effects by averaging all blood pressure measurements available in the obstetric files.

Although the association between blood lead levels and PIH persisted in the multivariate analysis, it is also possible that it reflects residual confounding due to unmeasured confounders. Geographic residence is a general covariate, and residual confounding by this variable cannot be ruled out. However, if this residual confounding explained the association found in the multivariate analysis, it is likely that an interaction between geographic residence and blood lead levels would have been observed. Moreover, blood lead concentration may be a better estimate of maternal environmental exposure than external indicators, and its use as a continuous variable in the regression model limits the risk of residual confounding.

PIH remains a multifactor disease with unclear etiology, which could compromise maternal reproductive and newborn outcomes. We identified a significant association between maternal blood lead levels in mid-pregnancy and blood pressure. Our findings suggest that lead may have an etiologic role in PIH, even at low levels of environmental exposure, and thus incite public health organizations to revise the upper limit of “acceptable” blood lead levels in pregnant women, which is currently at 10 μg/dL.

## Figures and Tables

**Table 1:** Baseline characteristics according to PIH occurrence among 971 pregnant women with no history of chronic hypertension.

Characteristic	PIH	No PIH	*p*-Value
Maternal age (%)			0.70

< 25 years	19.8	17.6	
25–34 years	64.2	68.2	
≥ 35 years	16.0	14.2	
*No.*	106	865	

Education (%)			0.87

≥ 12 years	64.2	66.2	
< 12 years	30.2	29.0	
Unknown	5.7	4.7	
*No.*	106	865	

Socioeconomic status (%)			0.76

High	25.7	25.5	
Medium	52.4	55.5	
Low	21.9	19.0	
*No.*	105	851	

Geographic residence (%)			< 0.001

Poitiers (western)	36.8	58.6	
Nancy (eastern)	63.2	41.4	
*No.*	106	865	

Hematocrit, (mean ± SD)^a^	35.2 ± 2.5	34.6 ± 2.6	0.02

*No.*	105	842	

BMI before pregnancy (%)			< 0.001

< 25	53.3	76.2	
25–29.9	24.8	17.5	
≥ 30	21.9	6.3	
*No.*	105	835	

Weight gain during pregnancy [kg (mean ± SD)]	13.4 ± 6.5	13.4 ± 4.6	0.92

*No.*	104	834	

Gestational diabetes (%)			< 0.001

No	83.0	94.1	
Yes	17.0	5.9	
*No.*	106	851	

Parity (%)			0.71

Nulliparous	42.5	44.3	
Multiparous	57.5	55.7	
*No.*	106	848	

Smoking during pregnancy (%)			0.86

0 cigarettes/day	72.6	71.7	
≥ 1 cigarette/day	27.4	28.3	
1–9 cigarettes/day	19.6	22.2	
≥ 10 cigarettes/day	7.8	6.1	
*No.*	102	820	

Alcohol consumption before pregnancy (%)			0.90

No	28.2	28.7	
Yes	71.8	71.3	
*No.*	103	849	

Iron/calcium supplementation (%)			0.95

No	2.8	3.9	
Yes	61.3	60.1	
Unknown	35.9	36.0	
*No.*	106	865	

Premature delivery [before 37 weeks (%)]			< 0.001

No	86.8	95.1	
Yes	13.2	5.0	
*No.*	106	851	

Neonate weight [g (mean ± SD)]	3126.7 ± 719.9	3299.0 ± 493.6	0.02

*No.*	106	844	

Neonate sex (%)			0.28

Male	58.5	52.9	
Female	41.5	47.1	

**Table 2:** Distribution level of elements in maternal blood at mid-pregnancy, according to PIH occurrence.

	PIH (*n* = 106)		No PIH (*n* = 865)		
Element	Mean ± SD	Geometric mean	Mean ± SD	Geometric mean	*p*-Value^a^
Lead (μg/dL)	2.2 ± 1.4 ^b^	1.9	1.9 ± 1.2	1.6	0.02
Cadmium (μg/L)	0.9 ± 0.5	0.8	0.9 ± 0.6	0.7	0.08
Manganese (μg/L)	11.8 ± 6.3	10.6	10.6 ± 4.5	9.6	0.06

**Table 3:** ORs for PIH, according to maternal blood lead distribution and overall outcome characteristics.

	Unadjusted analysis		Adjusted analysis^a^	
	
Quartile	OR (95% CI)	*p*-Value	OR (95% CI)	*p*-Value
Log (lead)^b^	3.49 (1.37–8.87)	0.009	3.29 (1.11–9.74)	0.03
Q1 (referent)	1.0		1.0	
Q2	1.55 (0.78–3.11)	0.94	1.84 (0.77–4.41)	0.84
Q3	1.61 (0.78–3.31)	0.81	2.07 (0.83–5.13)	0.50
Q4	2.19 (1.09–4.41)	0.06	2.56 (1.05–6.22)	0.09

Number of observations used in adjusted analysis: 720; convergence criterion satisfied; *R*^2^ = 15.7%. Quartiles of maternal blood lead distribution: Q1, < 1.20 μg/dL; Q2, 1.20–1.70 μg/dL; Q3, 1.71–2.30 μg/dL; Q4, > 2.30 μg/dL.

a Adjusted for maternal age; cadmium, manganese, and selenium blood levels; hematocrit; parity; BMI; gestational diabetes; educational level; socioeconomic status; geographic residence; and smoking status during pregnancy.

b Log-transformed maternal blood lead used as a continuous variable.

**Table 4:** ORs for PIH according to parity, per unit increase in blood lead level.

Measure	Nulliparous	Multiparous
PIH incidence (%)	10.7	11.4
Unadjusted OR (95% CI)	1.8 (0.5–6.6)	3.7 (1.1–12.3)
Adjusted OR^a^ (95% CI)	2.9 (0.6–15.7)	4.6 (1.0–21.6)

a Adjusted for maternal age; cadmium, manganese, and selenium blood levels; hematocrit; BMI; gestational diabetes; educational level; socioeconomic status; geographic residence; smoking status and alcohol consumption before pregnancy.
